# Ice matters: Life‐history strategies of two Antarctic seals dictate climate change eventualities in the Weddell Sea

**DOI:** 10.1111/gcb.15828

**Published:** 2021-09-07

**Authors:** Mia Wege, Leo Salas, Michelle LaRue

**Affiliations:** ^1^ Gateway Antarctica School of Earth and Environment University of Canterbury Christchurch New Zealand; ^2^ Department of Zoology & Entomology University of Pretoria Hatfield Pretoria South Africa; ^3^ Point Blue Conservation Sciences Petaluma CA USA; ^4^ Department of Earth and Environmental Sciences University of Minnesota Minneapolis MN USA

**Keywords:** breeding season, conservation management, crabeater seal, ensemble machine learning, Southern Ocean, species distribution modelling, Weddell Seal

## Abstract

The impacts of climate change in Antarctica and the Southern Ocean are not uniform and ice‐obligate species with dissimilar life‐history characteristics will likely respond differently to their changing ecosystems. We use a unique data set of Weddell *Leptonychotes weddellii* and crabeater seals' (CESs) *Lobodon carcinophaga* breeding season distribution in the Weddell Sea, determined from satellite imagery. We contrast the theoretical climate impacts on both ice‐obligate predators who differ in life‐history characteristics: CESs are highly specialized Antarctic krill *Euphausia superba* predators and breed in the seasonal pack ice; Weddell seals (WESs) are generalist predators and breed on comparatively stable fast ice. We used presence–absence data and a suite of remotely sensed environmental variables to build habitat models. Each of the environmental predictors is multiplied by a ‘climate change score’ based on known responses to climate change to create a ‘change importance product’. Results show CESs are more sensitive to climate change than WESs. Crabeater seals prefer to breed close to krill, and the compounding effects of changing sea ice concentrations and sea surface temperatures, the proximity to krill and abundance of stable breeding ice, can influence their post‐breeding foraging success and ultimately their future breeding success. But in contrast to the Ross Sea, here WESs prefer to breed closer to larger colonies of emperor penguins (*Aptenodytes forsteri*). This suggests that the Weddell Sea may currently be prey‐abundant, allowing the only two air‐breathing Antarctic silverfish predators (*Pleuragramma antarctica*) (WESs and emperor penguins) to breed closer to each other. This is the first basin‐scale, region‐specific comparison of breeding season habitat in these two key Antarctic predators based on real‐world data to compare climate change responses. This work shows that broad‐brush, basin‐scale approaches to understanding species‐specific responses to climate change are not always appropriate, and regional models are needed—especially when designing marine protected areas.

## Graphical abstract

We built habitat models for crabeater (CES) and Weddell (WES) seals using presence–absence data identified from satellite images. The models show that because the dietary specialist crabeater seals breed on pack ice close to where their preferred prey (Antarctic krill) is most abundant, they are at risk to lose breeding habitat. Weddell seals, being a dietary generalist who breed on more stable fast ice, are likely to buffer climate change effects.

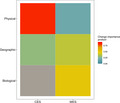

## 1 INTRODUCTION

Climate change will impact animal populations differently based on a combination of the latitude, baseline ecosystem stability and the life‐history strategy of a particular species, with ultimate impacts on global distribution and abundance of populations (e.g. Isaac, 2009; Mawdsley et al., 2009; Visser, 2008). For example, Southern Ocean temperatures in the Antarctic Peninsula region are some of the most rapidly warming on the planet (Ducklow et al., 2007) but directly east across the Peninsula itself in the Weddell Sea, comparatively little has changed with respect to sea ice concentration or extent since records have been kept (Parkinson, 2019), with minor decreases in summer extent observed only very recently (Turner et al., 2020). Reasonably, we would anticipate diverging responses by ice‐obligate species to such changes. Their responses will likely depend on regional sea ice habitat and behavioural plasticities that dictate their life history. Therefore, climate change‐induced fluctuations in populations across nearby locations could easily be very different. The implications for different regional‐specific population outcomes are pertinent to conservation and management priorities, as further human‐induced changes in the Southern Ocean include fishing for krill (*Euphausia* spp.) and other commercially important species such as fishes (e.g. Antarctic toothfish [*Dissostichus mawsoni*]). How do we plan for possible compensatory or additive changes to an ecosystem such as fishing and climate change? A first step is that we must understand the habitat associations and life histories of those marine predators that indicate the health of ocean ecosystems.

In the Southern Ocean, two closely related seal species segregate spatially through habitat associations, particularly in the breeding season. The Weddell seal (WES; *Leptonychotes weddellii*) is a mixed capital‐income breeding, long‐lived pinniped (Wheatley et al., 2008) that breeds on only ~0.55% of fast ice habitat along the Antarctic coastline during austral spring (LaRue et al., 2020) and return to the same pupping locations annually (Rotella et al., 2012). They also have a generalist approach to diet, which ranges across many species of fishes, cephalopods and crustaceans (Burns et al., 1998; Lake et al., 2003). Due to its association with fast ice habitat, the WES is probably in similar jeopardy because of climate change as the emperor penguin (Jenouvrier et al., 2020; Trathan et al., 2020). For both species, the decrease in fast ice extent would result in contracting distributions, putting the seals in possible competition for breeding and haul‐out space with the penguins (LaRue et al., 2019). Adding to the complexity is the Antarctic toothfish fishery, which has operated in the Ross Sea for >20 years and may be causing shifts in population distributions and/or a trophic cascade, of which the seals are part (Ainley et al., 2015).

In contrast, the crabeater seal (CES; *Lobodon carcinophagus*) has a specialist strategy in many regards. Though, a capital breeder and likely one of the most abundant marine mammals in the world (Laws, 1977), CESs are found almost exclusively in the pack ice, which they use as a platform for hauling out to rest, a place to forage from and a place to birth their young (Adam, 2005; Bengtson & Siniff, 1981). Heavy reliance on pack ice habitat dictates that the species has no population structure (e.g. no philopatry; (Curtis et al., 2011), and also explains their notably specialized diet of krill (*Euphausia* spp.) that live and breed mostly under the pack ice and pack ice edges comprises >90% of diet (Hückstädt et al., 2012). In fact, because CES dentition has evolved to filter krill out of seawater—a very specific evolutionary trait—the species is considered an important indicator of krill distribution and abundance in the Southern Ocean. Specialist foragers are thought to be more sensitive to fluctuations in food abundance and distribution (Angermeier, 1995; Shultz et al., 2005) because they might not be able to switch diet or change foraging habitat quickly enough to compensate for bottom‐up changes (Terraube et al., 2011). Over millennia, these seals have evolved to follow krill populations, which are often associated with pack ice; what might we expect if one or both (pack ice distributions or krill populations) change dramatically enough that the seals are required to expend more energy to forage?

Not all regions in Antarctica are experiencing climatic changes in the same way or at the same rate (Chown & Brooks, 2019). As a result, comparing climate influences on populations between species is difficult due to the added regional variation. Potential variation caused by regional differences needs to be removed to understand how species will respond to climate change. To date, such issues have been difficult to address due to the inaccessibility of the Southern Ocean habitats in which both of these species exist. However, with high‐resolution satellite imagery (VHR; 0.3–0.6 m spatial resolution; Gonçalves et al., 2020; LaRue et al., 2011; Wege et al., 2020), we can remotely view inaccessible, extensive pack ice and fast ice and therefore advance our understanding of seal habitat associations that underpin our ability to adequately predict the potential for future change. Here, we aimed to understand the various physical and biological factors that explain WES and CES breeding habitat in the Weddell Sea (LaRue et al., 2019; Wege et al., 2020). The Weddell Sea is one of the areas least affected by climate change in the Southern Ocean, making it the ideal study location to understand life‐history driven differences in habitat use rather than climate change induced. But because the vast areas of the Weddell Sea are covered by multi‐year sea ice, it is impossible to collect breeding season presence–absence data on both species. For the first time, satellite imagery enables us to create an evidence‐based habitat model comparison between two key Antarctic predators, comparing climate sensitivities between specialist and generalist predators during the critical life phase of breeding. Drawing on similar studies looking at climate sensitivities for marine mammals in the Arctic (Laidre et al., 2008), we build on the study by Siniff et al. (2008), to explain important habitat variables for each seal species. We discuss implications of a marine protected area (MPA) in one of the important climate refugia in the Southern Ocean (Jenouvrier et al., 2014; Teschke et al., 2020), the Weddell Sea.

## 2 METHODS

### 2.1 Study area

**FIGURE 1:**
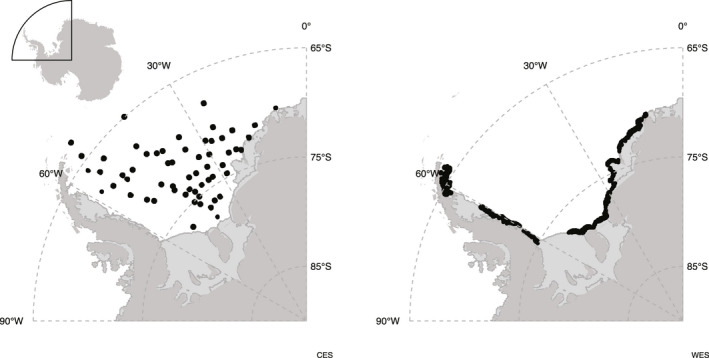
Locations of the satellite images used in this study for crabeater seals (CESs, left) and Weddell seals (WESs, right). The position of the Weddell Sea in relation to Antarctica is shown in the inset

We focused our efforts on the pack ice (CESs) and fast ice (WESs) habitats in the Weddell Sea (as in Wege et al., 2020; Figure 1), which is often defined by the Weddell Gyre and is situated roughly between 0°–60°W longitude and ~65°S until the ocean meets the continent at around 80°S at the southernmost end at the Ronne‐Filchner Ice Shelf. The Weddell Sea is characterized by exceptionally cold and deep waters, where Antarctic Bottom Water is formed (Carmack & Foster, 1975; Foster & Carmack, 1976), and sea ice conditions have changed relatively little since the onset of the Industrial Revolution. In fact, at the outer fringes of the Weddell Sea towards the Scotia Sea, the sea ice extent was at one point expanding (Bintanja et al., 2013; Meehl et al., 2016; Zhang, 2007) but is now observed to be decreasing (Turner et al., 2020). Other marine predators in the region include orcas (*Orcinus orca*), leopard seals (*Hydrurga leptonyx*), Adélie penguins (*Pygoscelis adeliae*; particularly in the north‐western portion), emperor penguins (*Aptenodytes forsteri*), Antarctic petrels (*Thalassoica antarctica*), snow petrels (*Pagodroma nivea*), South polar skua (*Catharacta maccormacki*; (Harris et al., 2015)), minke whales (*Balaenoptera bonaerensis*) and humpback whales (*Megaptera novaeangliae*). The Weddell Sea has some of the highest concentrations of primary productivity because of its relatively large sea ice edge where phytoplankton blooms occur as the ice melts (Moore & Abbott, 2000; Vernet et al., 2019). The southern reaches of the Weddell Sea has low Antarctic krill concentrations, whereas the Scotia Sea, just to the north, has the highest krill concentrations, and subsequent active fisheries, in the Southern Ocean (Atkinson et al., 2008). Antarctic toothfish are also abundant in the Weddell Sea and have been commercially exploited as a fishery on the northern edges of the Weddell Gyre (Roberts et al., 2011).

### 2.2 Environmental variables

We used remotely sensed environmental variables as predictors to build species‐specific habitat models. We chose variables based on common indicators that influence the breeding habitat of CESs and WESs respectively; given they differ (pack ice vs fast ice breeders), the suite of environmental variables also differed. For models of CES habitat, the 22 environmental variables broadly included variables of ocean depth, temperature, oceanography and ice conditions (see Appendix Table A1 for the full list). Models of WES habitat considered 19 environmental variables, which were categorically similar but differentiated slightly: for example, fast ice conditions, proximity to penguin colonies, bathymetry (meanbathy), distances to glacier (glacierdist) and to the continent Antarctica (distToShore) and distances to the 300 m (cont300dist) and 800 m (cont800dist) bathymetry contours (Appendix Table A2). Because most oceanographic and ice variables are inherently collinear, we filtered for the most informative set by calculating variable inflation factors for the entire Weddell Sea region using the R library ‘fmsb’ (Nakazawa, 2018). Variables with a variance inflation factor larger than 10 were excluded, because this is a good indication of strong collinearity (Nakazawa, 2018).

### 2.3 Habitat modelling

We leveraged already‐published data sets on CES and WES presences identified from VHR imagery, during their respective breeding seasons in the Southern Ocean via Wege et al. (2020) and LaRue et al. (2020). To carry out habitat modelling and to determine the relative importance of environmental variables, we constructed separate habitat models for each species (i.e. one model where probability of presence of CESs was the response variable and one model where WES presence/absence was the response variable). We built habitat ensemble models, replicating methods in the study by Wege et al. (2020) which used Random Forests (RF), Boosted Regression Trees (BRT) and Maxent models. In R (with libraries, ‘caret’, ‘ranger’, ‘gbm’ and ‘ENMeval’; Kuhn et al., 2019; Muscarella et al., 2014; Ridgeway, 2015; Wright & Ziegler, 2017). We excluded the Support Vector Machine model from the ensemble used in the study by Wege et al. (2020), because it was the poorest performing of the three, and did not add value to the WES models (see below). We therefore opted to only use an ensemble of RF, BRT and Maxent models.

The full modelling procedure is detailed in the study by Wege et al. (2020), so here we provide a brief summary. For all CES and WES models, we held out 20% of the data (test data, *n*_CES_ = 1964 records; *n*_WES_ = 76 records) to evaluate model performance, while the remaining 80% of the data were used to train the models. The hold‐out data had equal numbers of seal presence and absence points (*n*_CES_ = 982 records, *n*_WES_ = 38) and were selected randomly across the entire study region. To compensate for the unbalanced number of seal presence versus seal absence records for both species (Wege et al., 2020), we made use of a bootstrapping method and ran each of the three model types (RF, BRT and Maxent) on equally sized subsets of the data. This also reduces the amount of spatial autocorrelation often found in habitat models (Hijmans, 2012). Each bootstrap contained all the seal presence records (*n*_CES_ = 3929; *n*_WES_ = 134) and a random sample of equal size (*n*_CES_ = 3929; *n*_WES_ = 134) of seal absence records. Without balancing the data set, the models would optimize on predicting absences well at the expense of increased error in predicted presence. We ran 500 bootstraps for each of the three constituent models for each species. The RF and BRT models were tuned, respectively, in each bootstrap using the ‘tuneGrid’ function in ‘caret’ through compiling a range of candidate models and choosing the best candidate model based on the *R* ^2^ value that was calculated from 10‐fold cross‐validation. Cross‐validation folds were created by randomly dividing bootstrap records into 10‐folds containing an approximately equal number of records. We calculated the *R* ^2^ value as the goodness‐of‐fit measure for the RF and BRT models of each bootstrap using the 20% hold‐out test data.

### 2.4 Variable importance

The main aim was to identify environmental variables that influence CES and WES breeding habitat. For each of the bootstrap samples, we calculated permutation variable importance for the RF and Maxent models and percentage contribution of each variable in the BRT models. These methods differ with respect to the way variable importance is calculated, but the form and magnitude of important explanatory variables are comparable among them. To enable us to compare permutation importance with percentage relative influence values produced by the different models, we calculated the relative proportion each of these variables contributed to seal breeding habitat.

Returning to methods outlined in the studies by Laidre et al. (2008) and Siniff et al. (2008), we then used the quantitative results of variable importance to inform and group sensitivities of each species’ life history to changes in the environment. Based on what is currently known for Southern Ocean climate change impacts from decades‐worth of literature and based on experiences of the authors, all authors independently ranked whether the variable is likely to change from 0 to 3 with 0 being no change, 1 = low level of change, 2 = medium level of change and 3 = high level of change; scores were then averaged for each variable creating a ‘climate change score’. For example, distance to the ice edge was ranked as level 3, because we know increasing sea temperatures will cause (and are already causing) sea ice to melt and recede; but bathymetry is given a score of 0, because the likelihood of change is nil. We then calculated a variable we called the ‘*change importance product*’, which is the climate change score multiplied by the proportional importance of the variable for each of the three model types (see above paragraph). This was done to come up with a relative index that considers the importance of the environmental variable for each species’ life history and the likelihood of change of that aspect of the species' environment. The aim here was to distinctly separate variables that are both important and also likely to change substantially, from less important or more stable environmental situations for each species.

## 3 Results

### 3.1 Variance inflation factors

The Variance inflation factors of the CES data indicated high collinearity between 10 variables and consequently, only 14 of the 24 variables were retained for habitat modelling: bathy, dist_canyon, dist_shelf, ice_edge_dist, ice_sd, oldice_cv, sal200_600, slope, vmix, vmix_sd, windmag, sst, shflux and shflux_sd. Variance inflation factors calculated for the WES data indicated high collinearity for two variables. Subsequently, 17 of the 19 variables were retained: ADPEabund, ADPEdist, cont300dist, cont800dist, DecemberIcePresence, distNearestIceEdge, distToShore, EMPEabund, EMPEdist, fastIceRatio, glacierdist, InTrough, meanbathy, meanslope, Persistence2Years, PredictabilityDec5Years and PredictabilityOct5Years.

### 3.2 Model performance

As in the study by Wege et al. (2020), the CES RF and BRT models had low overall performance (Table 1), but these models were validated through agreement with predicted breeding habitat using Maxent models that performed better. The WES RF and BRT models predicted WES breeding habitat with 94% accuracy (Table 1), while the Maxent models’ average was 86% accuracy compared with 76% accuracy for the CES Maxent models (Table 1).

**TABLE 1:** Model goodness‐of‐fit metrics for each of the models and species. Continuous predictors provide an *R* ^2^, while binomial presence–absence data goodness of fit metric is the area under the curve (AUC) of the receiver operating characteristic curve

Model	CES	WES
BRT	*R* ^2^ = 0.086 ± 0.0008	AUC = 0.94 ± 0.01
RF	*R* ^2^ = 0.076 ± 0.002	AUC = 0.94 ± 0.009
Max	AUC = 0.71 ± 0.004	AUC = 0.86 ± 0.010

### 3.3 Variable importance

**FIGURE 2:**
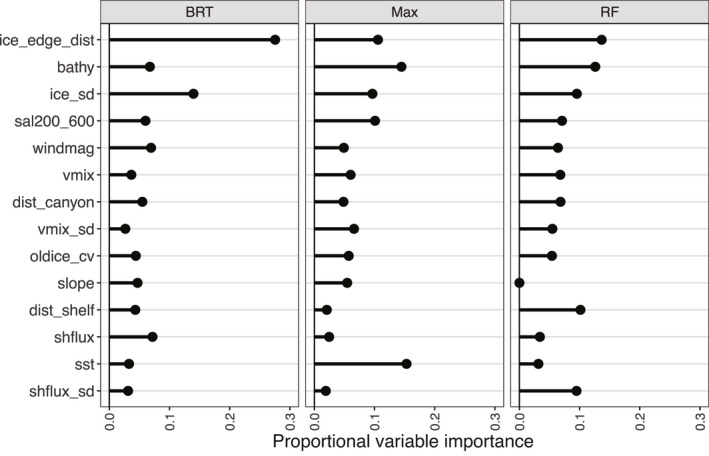
Crabeater seals: Proportional variable importance for the 500 bootstraps of the Random Forest (RF), Boosted Regression Tree (BRT) and Maxent (Max) models

**FIGURE 3:**
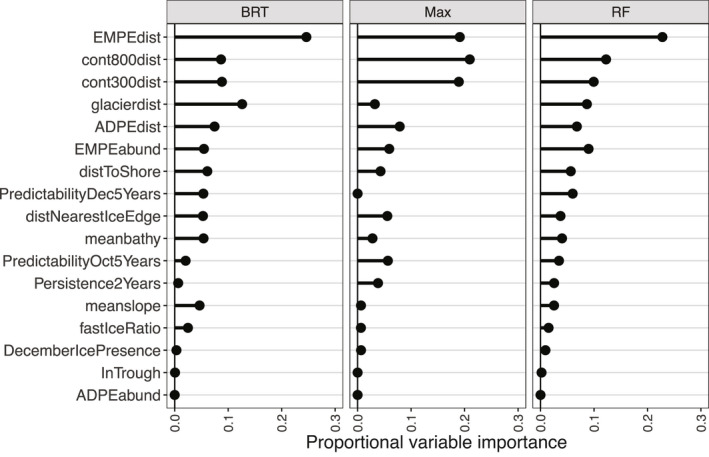
Weddell seals: Proportional variable importance for the 500 bootstraps of the Random Forest (RF), Boosted Regression Tree (BRT) and Maxent (Max) models

**FIGURE 4:**
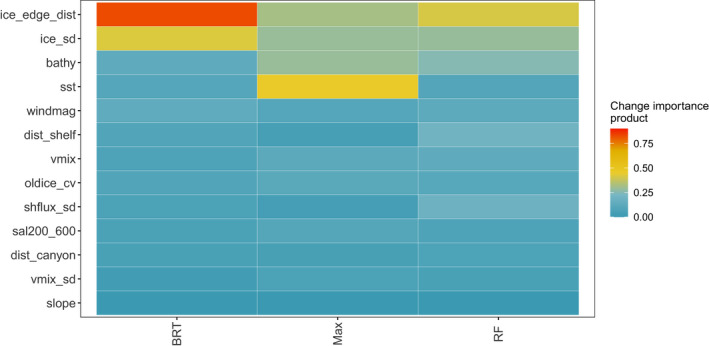
Crabeater seal models' change importance product (the product of the variable proportional importance and estimated rate of change) for each variable per model. Variables are ranked by their overall mean change importance product value in a descending order

**FIGURE 5:**
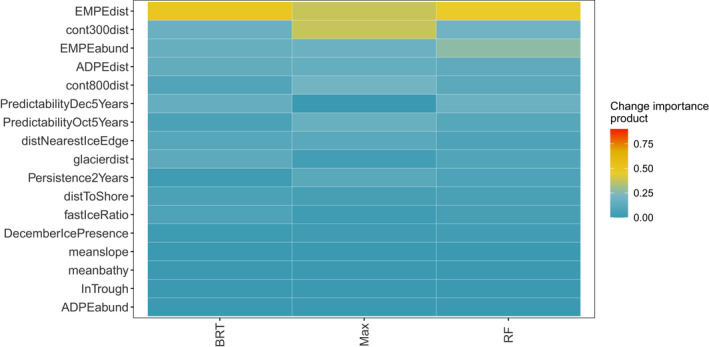
Weddell seal models' change importance product (the product of the variable proportional importance and estimated rate of change) for each variable per model. Variables are ranked by their overall mean change importance product value in a descending order

**FIGURE 6:**
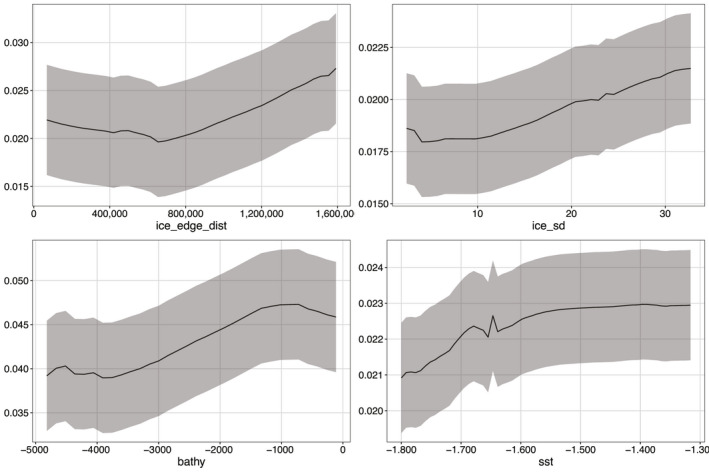
Crabeater seal partial dependence plots showing how the probability of seal presence (±SD across the models and bootstraps) is influenced by the top four environmental predictor variables. Image taken from the study by Wege et al. (2020). ice_edge_dist – distance from the ice edge (m), bathy – bathymetry (m), ice_sd – ice concentration standard deviation and dist_shelf – distance to the continental shelf (m)

**FIGURE 7:**
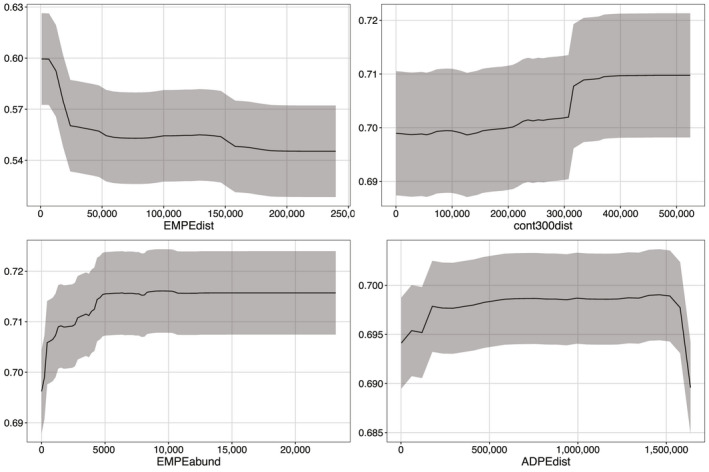
Weddell seal partial dependence plots showing how the probability of seal presence (±SD across the models and bootstraps) is influenced by the top four environmental predictor variables. EMPEdist – distance to emperor penguin colony (m), cont300dist – distance from the 300‐m depth contour (m), glacierdist – distance from a glacier (m) and ADPEdist – distance to Adélie penguin colony (m)

The CES RF, BRT and Maxent top variables across all models were as follows: distance to ice edge, bathymetry, standard deviation of ice concentration and distance to the shelf (Figure 2). For WESs, top variables that agreed across all models were as follows: distance to emperor penguin colonies, distances to 300 and 800 m bathymetric depths and distance to glaciers (Figure 3). When incorporating the climate change score, the variables most influencing CES presence were distance to ice edge, standard deviation of ice concentration, bathymetry and sea surface temperature (Figure 4). Weddell seal presence, when taking into account the climate change score, was most influenced by penguin colony‐associated variables and proximity to ocean depths of 300 m. Interestingly, distance to glaciers dropped down the order, while predictability of ice in December and October over the last 5 years increased (Figure 5). Overall, the CES change importance product ranged between 0 and 0.83; while WES change importance product scores ranged between 0 and 0.49 (see Table S3 for all change importance product scores). Figures 6 and 7 show the relationship between the top six variables based on the change importance product score for each species, respectively, and the likelihood for seal presence, rather than the top variables based only on variable importance.

## 4 DISCUSSION

More than 10 years ago, Siniff et al. (2008) discussed the potential fates of Antarctic seal species due to climate change. Based on an arbitrary scale of potential effects (ranging from −4 to 4) and expert opinion, authors found that both CESs and WESs Antarctica‐wide would likely be most negatively impacted by losses in sea ice and by the fishing industry. Specifically, they suggested that CESs are unlikely to prey‐switch sufficiently to make up for krill loss, and that WESs would be affected by ice variables during the pup‐rearing season (either a loss of fast ice or increased fast ice thickness). While Siniff et al. (2008) focused broadly on human‐induced physical changes to the entire Southern Ocean system, we quantified fine‐scale habitat variables (including ecological influences such as penguin colony presence and abundance), qualitatively assessed likely change, and combined and compared these relative results among two sympatric, ice‐obligate species during their rarely studied breeding season (Figures 2 and 3). To our knowledge, this work is the first to carry out such a comparison in an effort to inform future, detailed conservation and management decisions.

We found that the most influential variables for CESs, that are also likely to change the most in the future, are connected to their ability to forage effectively post‐breeding. Crabeater seals are capital breeders that do not forage during their breeding season (Bengtson & Stewart, 1992). This is critical, as CESs are one of the most specialized predators and largest consumers of krill in the Southern Ocean (Hill et al., 2006; Laws, 1977) and are unlikely to prey‐switch to make up energetic losses due to loss and spatial shifts of krill (Hückstädt et al., 2020). As in the study by Wege et al. (2020), CES breeding distribution was dictated by proxies for either distribution of krill or physical aspects of pack ice habitat (distance to the ice edge, variation in ice concentration, bathymetry and distance to the continental shelf). We suggest CESs prefer to breed close to or over their prey source, likely due to their highly specialized diet and because they are not central‐place foragers bound to a central colony location. When CESs end their breeding haul out and fasting that goes with it, they need to replenish their body reserves quickly to remain successful breeders in the following year (Boyd, 2000; McDonald et al., 2008), but the ephemeral and ever‐changing nature of pack ice could restrict the movements of seals and prevent them from reaching productive krill patches in open water, which is why higher ice concentration variability was preferred (Figure 6). Crabeater seals need stable ice to breed on, but at the same time in the mostly ice‐covered Weddell Sea, they require cracks and leads to open up regularly to provide pathways through which they can access open water fast and easily. However, taking the likelihood of climate change impacts on each of the modelled variables into account, we found sea surface temperature replaced distance to the continental shelf in its importance to the future of CES distributions in the Weddell Sea, meaning that rising sea surface temperatures could result in the ultimate loss of a platform to breed that is stable and large enough to last the entire breeding season.

This spatial overlap between resting and foraging habitat also happens outside the breeding season and can therefore be used as a proxy for krill distribution (Hückstädt et al., 2020). In the western Antarctic Peninsula, changing climate is affecting the ecosystem at an alarming rate (Vaughan et al., 2003) through changes in circulation, sea ice distribution and water temperatures. Because of these changes in ocean properties, prey species of CESs will shift further offshore and CESs will expand their distribution to offshore waters in response to prey distribution shifts (Hückstädt et al., 2020). Seals will end up either incurring higher energetic costs while travelling further to profitable foraging areas, or forage on lower concentrations of krill or switch their preferred prey resource (Hückstädt et al., 2020).

Although climate change responses by krill are still debated, some evidence suggests krill habitat is predicted to have a lower krill growth potential, due to an increase in sea surface temperature and chlorophyll‐a concentration (Veytia et al., 2020). This may cause a disconnect between krill's annual cycle and the future environment (Veytia et al., 2020). Additionally, krill abundance and recruitment rate to adulthood are currently influenced by increasing sea temperatures and reduction in winter sea duration both caused by climate change (Flores et al., 2012), while increasing temperatures and subsequent ocean acidification will decrease hatch rate in the Atlantic sector of the Southern Ocean (Kawaguchi et al., 2013).

While other ice‐obligate species are seemingly capable of behavioural changes given environmental shifts (Bestley et al., 2020) (e.g. WESs [Chambert et al., 2015]; emperor penguins [Fretwell et al., 2014], Adélie penguins [Dugger et al., 2010]), there is no evidence to suggest CESs have the capability to adapt in the same way. We thus caution against arguments that suggesting climate change impacts may supersede any food web alterations in the Weddell Sea and immediately surrounding seas. Indeed, CES abundance in the Weddell Sea during spring was observed to have declined as early as the end of the previous century, 1960–1990s (Joiris, 1991). In addition, circumstantial evidence suggests spring pack ice densities remain lower than before in the region (Bester et al., 2021). Thus, any future marine protection will likely require measures that take into consideration not only loss of habitat due to climate change, but also the loss of critical prey and changes in prey distribution that may ultimately impact foraging efficiency. The Atlantic sector of the Southern Ocean—north of the Weddell Sea embayment—has the highest abundances of krill (Atkinson et al., 2008), making our understanding of CES distribution and potential future impacts so much more important.

Comparatively, distributions of the WES, the fast ice obligate, in the Weddell Sea were influenced by distances to emperor penguin colonies, glaciers and to the 300 m and 800 m bathymetrical contour, somewhat similar to findings in the Ross Sea (LaRue et al., 2019). However, when accounting for likelihood of climate change influences, biological factors such as abundance of and distance to both penguin species’ colonies overtook physical habitat variables such as distance to glaciers and ocean depth (Figures 3 and 5). This suggests that as the climate continues to warm and sea ice becoming increasingly important as a scarce and limiting resource, the overarching influence for WESs may be their potential competition with penguins for resources. In this case, all three species are likely to compete for Antarctic silverfish (*Pleuragramma antarctica*), an important food item in all three species’ diets (Ainley, 2002; Burns & Kooyman, 2001). LaRue et al. (2019) suggested a potential relationship between seals and penguins in the Ross Sea (the largest MPA in the world), where generalized linear modelling for seal presence suggested that over the entire region, not only was deep water (>300 m depth) important, but proximity to smaller emperor penguin colonies and increasing distances from Adélie penguin colonies were critical.

In contrast to the Ross Sea (LaRue et al., 2019), here we found WESs preferred to be nearby where emperor penguin colonies were larger. We suggest this may be due to the differences in community ecology between the two regions. In the meso‐predator dense Ross Sea, where all three species (both penguins and WESs) are in high abundance and their colonies in relatively close proximity to each other (LaRue et al., 2019; Lynch & LaRue, 2014; Santora et al., 2020), there may be different ecological relationships at play that are absent in the Weddell Sea. For example, millions of Adélie penguins breed throughout the Ross Sea over dozens of colony locations making up ~33% of the global population (Lynch & LaRue, 2014), whereas emperor penguins exist in seven colonies with <100,000 birds in total (Fretwell et al., 2012) and WESs are found mostly south of the Drygalski Ice Tongue (LaRue et al., 2019) where penguins are largely absent; this orientation suggests a possible spatial segregation among the penguins and seals. In the Ross Sea, there is evidence for a relationship between subregional effects and seal distribution (LaRue et al., 2019). In addition, there are likely to be intra‐ and interspecific effects between the two penguin species (e.g. emperor penguin colonies are distributed uncannily equidistant from each other, whereas Adélie penguin colonies occur in clusters; Santora et al., 2020). In the Weddell Sea, Adélie penguin colonies are found only in the north‐western tip of the region, whereas fast ice obligate seals and emperor penguins are the only sympatric air‐breathing Antarctic silverfish predators. In other words, if WESs and penguins do compete, seals possibly have less competition from penguins in the Weddell Sea compared to the Ross Sea.

If emperor penguins and WESs do indeed compete or niche partition, perhaps the minor difference among explanatory variables dictating WES presence here (compared to the Ross Sea) is due to the Weddell Sea being prey‐abundant for these fast ice obligates. These community ecological effects, rather than physical properties of the ocean, are likely to be the most important for WESs in the future, which is a critical finding for the future of conservation. Simply put, really only two air‐breathing Antarctic silverfish predators (WESs and emperor penguins) that can dive to the same depths (Goetz et al., 2018) are coexisting in space and time (i.e. pup‐ and chick‐rearing) in the Weddell Sea. WESs are notoriously generalist predators (Burns & Kooyman, 2001; Lake et al., 2003) and changes in ice conditions could change the dynamics of community ecology in the Weddell Sea via encroaching competitors, prey‐switching and/or decreases in prey. Divergent foraging strategies that exist among different individuals within a species, such as those observed by Photopoulou et al. (2020) for WESs in the Weddell Sea, also has the potential to result in divergent responses to climate effects. In general, our results support Siniff et al. (2008) suggesting that WESs are slightly buffered from additive impacts of climate and ecological shifts, compared to CESs. Notably, however, here we did not consider the direct effects a toothfish fishery could have on WESs in this region. The Weddell Sea is currently a region where a proposal for an MPA has been debated at the Commission for the Conservation of Antarctic Marine Living Resources (CCAMLR; Teschke et al., 2019). Conservation in the region needs to consider the effects of climate for the CES, the potential to alter community ecology for fast ice obligates, and finally to possibly put WESs in direct competition with humans for energetically important toothfish.

We note that although the error margins around the partial dependence plots (Figures 6 and 7) seem wide, these result from 500 bootstraps for three different types of models (BRT, RF and Maxent)—totalling 1500 bootstraps. Considering that model has its own nuanced way of estimating the relationship of species presence to the variable of interest, across 500 bootstraps there was notably a high degree of agreement for the most important variables to those models. Additionally, because these partial dependence plots include the uncertainty in the sample (i.e. variation among bootstrap samples), model uncertainty (i.e. predictive differences among models) and prediction error due to error intrinsic in the data, we note that there were still very clear patterns. Most importantly, our models were trained without any constraints about the functional form of these relationships. Yet, these results fully agree with our expectation based on the known biology of the species. For example, the CES models all clearly pointed to variables that influence adult krill distribution, the prey species that make up 90% of their diet.

A primary objective in conservation biology is the maintenance of biodiversity (Niesenbaum, 2019) and in the Southern Ocean in particular, conservation includes ecosystem maintenance—rather than single‐species protections—which is paramount to conservation planning (Gutt et al., 2020). Understanding the life‐history traits of animals provides a foundation for gauging population responses to environmental change, allowing us to mitigate and plan for potential shifts in distribution or abundance; thus, attempting to ensure ecosystem function and biodiversity. Such detailed understanding is particularly important because there will be winners and losers as anthropogenic climate change continues to warm polar environments, and the potential additive complexity of resource extraction exacerbates the problems in understanding how species and ecosystems may respond. A detailed understanding about ice‐obligate species’ responses to climate or resource extraction across regions is required to ensure adequate protections for biodiversity and ecosystem function.

## Supporting information

Supplementary Material

## Data Availability

The data that support the findings of this study are available from the corresponding author upon reasonable request.
