# Supplementary material for: Ice matters: Life‐history strategies of two Antarctic seals dictate climate change eventualities in the Weddell Sea
Source: Glob Chang Biol. 2021 Sep 7;27(23):6252–62. doi: 10.1111/gcb.15828 (PMC9293148; doi:10.1111/gcb.15828)
Supplement: Supplementary file 1 — Supplementary Material [file GCB-27-6252-s001.docx]

**Appendix:**

**Table A1: Environmental variables used as covariates in habitat suitability models for crabeater seals (*Lobodon carcinophaga*) in the Weddell Sea.**

| **Variable** | **Unit** | **Data Source** | **Temporal resolution** | **Original spatial resolution** |
| --- | --- | --- | --- | --- |
| Bathymetry (bath) | m | General Bathymetric Chart of the Oceans (British Oceanographic Data Centre) http://www.gebco.net | NA | 0.02° |
| Ocean floor slope (slope) | ° | Bathymetry derivative | NA | 0.02° |
| Sea surface temperature (sst) | °C | National Centres for Environmental Information https://www.ncdc.noaa.gov/oisst | Monthly | 0.25° |
| Sea surface temperature anomalies (sstA) | °C | Sea surface temperature derivative | Monthly | 0.25° |
| Sea surface temperature gradient (sst_grad) | ° | Sea surface temperature derivative | Monthly | 0.25° |
| Sea surface height (ssh) | m | Ssalto/Duacs (Copernicus Marine and Environment Monitoring Service) http://marine.copernicus.eu | Daily | 0.25° |
| Horizontal geostrophic current magnitude (currmag) | cm/s | Ssalto/Duacs (Aviso and Centre national d'études spatiales) http://www.aviso.altimetry.fr/duacs/ | Weekly | 0.25° |
| Eddy kinetic energy (eke) Calculated as eke = 0.5(curru2 + currv2) | cm²/s² | Ssalto/Duacs (Aviso and Centre national d'études spatiales) http://www.aviso.altimetry.fr/duacs/  Horizontal geostrophic current velocity (curru)  Vertical geostrophic current velocity (currv) | Weekly | 0.25° |
| Horizontal wind magnitude (windmag) | m/s | National Centres for Environmental Information http://www.esrl.noaa.gov/psd/ | Daily | 1.9° |
| Distance to continental shelf – i.e. the 500 m isobath (dist_shelf) | m | Bathymetry derivative https://data.aad.gov.au/metadata/records/Polar_Environmental_Data  Distance to nearest area of sea floor of depth 500m or less. Processing steps: Distances calculated in km using the Haversine formula on a spherical earth of radius 6378.137km. Points in less than 500m of water (i.e. over the shelf) were assigned negative distances. | NA | 0.02° |
| Distance to nearest polynya  (dist_polynya) | m | https://data.aad.gov.au/metadata/records/Polar_Environmental_Data  Source data: AMSR-E satellite estimates of daily sea ice concentration at 6.25km resolution  Pixels that were (on average) covered by sea ice for less than 35% of the year were identified. The distance from each grid point on the 0.1-degree grid to the nearest such polynya pixel was calculated in km using the Haversine formula on a spherical earth of radius 6378.137km. (NB the threshold of 35% was chosen to give a good empirical match to the polynya locations identified by Arrigo and van Dijken (2003), although the results were not particularly sensitive to the choice of threshold. | NA | 6.26 km |
| Distance to canyon (dist_canyon) | km | https://data.aad.gov.au/metadata/records/Polar_Environmental_Data  Distance to the axis of the nearest canyon. Source data: O'Brien and Post (2010) seafloor geomorphic feature dataset, expanded from O'Brien et al. (2009). Mapping based on GEBCO contours, ETOPO2, seismic lines. Processing steps: Distances to nearest canyon axis calculated in km using the Haversine formula on a spherical earth of radius 6378.137km. Reference: O'Brien, P.E., Post, A.L., and Romeyn, R. (2009) Antarctic-wide geomorphology as an aid to habitat mapping and locating vulnerable marine ecosystems. CCAMLR VME Workshop 2009. Document WS-VME-09/10 | NA | 0.1° |
| Ice concentration (ice) | % | NSIDC concentration data, processed by the SMMR/SSMI NASA Team  http://nsidc.org/data/docs/daac/nsidc0051_gsfc_seaice.gd.html | Daily | 12.5 km |
| Distance to ice edge  (dist_ice_edge) | m | Ice concentration derivative: the shortest distance (metres) to the sea ice contour where ice concentration is <15%. | Daily | 12.5 km |
| Ice standard deviation (ice_sd) | NA | Ice concentration derivative: standard deviation was calculated as the variation in ice concentration over the breeding season: September to November of 2017. | 3 month composite | 12.5 km |
| Ice thickness  (ice_thick) |  | https://data.aad.gov.au/metadata/records/Polar_Environmental_Data | 7 year composite | 0.1° |
| Old ice  (oldice) | % | <https://data.aad.gov.au/metadata/records/Polar_Environmental_Data>  Proportion of time the ocean is covered by sea ice of concentration 85% or higher.  Source data: AMSR-E satellite estimates of daily sea ice concentration at 6.25 km resolution  Processing steps: Concentration data from 1-Jan-2003 to 31-Dec-2010 used. The fraction of time each pixel was covered by sea ice of at least 85% concentration was calculated for each pixel in the original (polar stereographic) grid. Data then regridded to 0.1-degree grid using triangle-based linear interpolation.  Reference: Spreen, G., L. Kaleschke, and G. Heygster (2008), Sea ice remote sensing using AMSR-E 89 GHz channels, J. Geophys. Res., doi:10.1029/2005JC003384 https://seaice.uni-bremen.de/sea-ice-concentration/ | 7 year composite | 0.1° |
| Old ice coefficient of variation  (oldice_cv) | NA | A derivative from the old ice layer. The coefficient of variation was calculated using the data and time scale mentioned above. | 7 year composite | 0.1° |
| Surface heat flux (shflux) | W/m^2^ | https://data.aad.gov.au/metadata/records/Polar_Environmental_Data | 3 month composite | 0.1° |
| Surface heat flux standard deviation (shflux_sd) | NA | https://data.aad.gov.au/metadata/records/Polar_Environmental_Data | 3 month composite | 0.1° |
| Vertical mixing  (vmix) | m/s | https://data.aad.gov.au/metadata/records/Polar_Environmental_Data | 3 month composite | 0.1° |
| Vertical mixing standard deviation (vmix_sd) | NA | https://data.aad.gov.au/metadata/records/Polar_Environmental_Data | 3 month composite | 0.1° |
| Salinity difference between 200 m and 600 m depth (sal200_600 | psu | Source data: World Ocean Atlas 2013 version 2 (National Oceanographic Data Center, Silver Springs, MD, U.S.A.)  <https://www.nodc.noaa.gov/OC5/woa13/>  Processing steps: Data from September to November 2017 were averaged, regridded to 0.1-degree grid using bilinear interpolation and the salinity difference between 200 m and 600 m depth was calculated. | 3 month composite | 0.25° |
| Salinity difference between 0 m and 600 m depth (sal0_200) | psu | Source data: World Ocean Atlas 2013 version 2 (National Oceanographic Data Center, Silver Springs, MD, U.S.A.)  <https://www.nodc.noaa.gov/OC5/woa13/>  Processing steps: Data from September to November 2017 were averaged, regridded to 0.1-degree grid using bilinear interpolation and the salinity difference between the surface (0m) and 200m depth was calculated. | 3 month composite | 0.25° |

**Table A2: Environmental variables used as covariates in habitat suitability models for Weddell seals (*Leptonychotes weddellii*) in the Weddell Sea.**

| **Variable** | **Unit** | **Data Source** | **Original spatial resolution** |
| --- | --- | --- | --- |
| Bathymetry (meanbathy) | m | General Bathymetric Chart of the Oceans (British Oceanographic Data Centre) http://www.gebco.net | 0.02° |
| Ocean floor slope (meanslope) | ° | Bathymetry derivative | 0.02° |
| Distance to glacier (glacierdist) | m |  | 0.25° |
| Distance to Antarctica  (distToShore) | m | Bathymetry derivative | 0.25° |
| Distance to the 300 m bathymetrical contour  (cont300dist) | m | Bathymetry derivative | 0.25° |
| Distance to the 800 m bathymetrical contour  (cont800dist) | m | Bathymetry derivative | 0.25° |
| Is the grid cell over a oceanic trough  (InTrough) | Yes/No | Bathymetry derivative | 0.1° |
| Presence of ice in the preceding December  (DecemberIcePresence) | presence/ absence | Derivative of ice data from The U.S. National Ice Center (NIC) <https://nsidc.org/> | 0.25° |
| Persistence of ice in the last 2 years in the grid cell  (Persistence2Years) | 0-2 years | Derivative of ice data from The U.S. National Ice Center (NIC) <https://nsidc.org/> | 0.25° |
| Predictability of ice during December in the last 5 years (PredictabilityDec5Years) | 0-5 years | Derivative of ice data from The U.S. National Ice Center (NIC) <https://nsidc.org/> | 1.9° |
| Persistence of ice in the last 3 years in the grid cell  (Persistence3Years) | 0-3 years | Derivative of ice data from the The U.S. National Ice Center (NIC) <https://nsidc.org/> | 0.25° |
| Predictability of ice during October in the last 5 years  (PredictabilityOct5Years) | 0-5 years | Derivative of ice data from The U.S. National Ice Center (NIC) <https://nsidc.org/> | 0.25° |
| Distance to the nearest ice edge  (distNearestIceEdge) | m | Derivative of ice data from The U.S. National Ice Center (NIC) <https://nsidc.org/> | 0.02° |
| Ratio of fast ice  (fastIceRatio) | 0-1 | Derivative of ice data from The U.S. National Ice Center (NIC) <https://nsidc.org/> |  |
| Fast ice width  (fastIceWidth) | m | Derivative of ice data from The U.S. National Ice Center (NIC) <https://nsidc.org/> | 6.26 km |
| Distance to the nearest Adélie penguin colony  (ADPEdist) | m | (Ainley et al., 2015; Lynch & LaRue, 2014) | 0.1° |
| Abundance/ size of the nearest Adélie penguin colony  (ADPEabund) | Nr of penguins | (Ainley et al., 2015; Lynch & LaRue, 2014) | 12.5 km |
| Distance to the nearest emperor penguin colony  (EMPEdist) | m | (Fretwell et al., 2014) | 12.5 km |
| Abundance/ size of the nearest emperor penguin colony  (EMPEabund) | Nr of penguins | (Fretwell et al., 2014) | 12.5 km |

Footnote: All code and data for these variables can be found at: https://github.com/leosalas/FastIceCovars

Table A3: Change product importance scores from the species-specific habitat models, for each model and variable.

| **Species** | **Variable** | **Group** | **Mean proportional**  **variable importance** | **Change Score** | **Change Importance Product** |
| --- | --- | --- | --- | --- | --- |
| Weddell | ADPEabund | BRT | 0.00 | 2 | 0.00 |
| Weddell | ADPEabund | Max | 0.00 | 2 | 0.00 |
| Weddell | ADPEabund | RF | 0.00 | 2 | 0.00 |
| Weddell | ADPEdist | BRT | 0.07 | 2 | 0.15 |
| Weddell | ADPEdist | Max | 0.08 | 2 | 0.16 |
| Weddell | ADPEdist | RF | 0.07 | 2 | 0.14 |
| Weddell | cont300dist | BRT | 0.09 | 2 | 0.18 |
| Weddell | cont300dist | Max | 0.19 | 2 | 0.38 |
| Weddell | cont300dist | RF | 0.10 | 2 | 0.20 |
| Weddell | cont800dist | BRT | 0.09 | 1 | 0.09 |
| Weddell | cont800dist | Max | 0.21 | 1 | 0.21 |
| Weddell | cont800dist | RF | 0.12 | 1 | 0.12 |
| Weddell | DecemberIcePresence | RF | 0.01 | 3 | 0.03 |
| Weddell | DecemberIcePresence | BRT | 0.00 | 3 | 0.01 |
| Weddell | DecemberIcePresence | Max | 0.01 | 3 | 0.02 |
| Weddell | distNearestIceEdge | BRT | 0.05 | 2 | 0.11 |
| Weddell | distNearestIceEdge | Max | 0.06 | 2 | 0.11 |
| Weddell | distNearestIceEdge | RF | 0.04 | 2 | 0.07 |
| Weddell | distToShore | BRT | 0.06 | 1 | 0.06 |
| Weddell | distToShore | Max | 0.04 | 1 | 0.04 |
| Weddell | distToShore | RF | 0.06 | 1 | 0.06 |
| Weddell | EMPEabund | BRT | 0.05 | 3 | 0.16 |
| Weddell | EMPEabund | Max | 0.06 | 3 | 0.18 |
| Weddell | EMPEabund | RF | 0.09 | 3 | 0.27 |
| Weddell | EMPEdist | RF | 0.23 | 2 | 0.46 |
| Weddell | EMPEdist | Max | 0.19 | 2 | 0.38 |
| Weddell | EMPEdist | BRT | 0.25 | 2 | 0.49 |
| Weddell | fastIceRatio | BRT | 0.02 | 3 | 0.07 |
| Weddell | fastIceRatio | Max | 0.01 | 3 | 0.02 |
| Weddell | fastIceRatio | RF | 0.02 | 3 | 0.05 |
| Weddell | glacierdist | BRT | 0.13 | 1 | 0.13 |
| Weddell | glacierdist | Max | 0.03 | 1 | 0.03 |
| Weddell | glacierdist | RF | 0.09 | 1 | 0.09 |
| Weddell | InTrough | BRT | 0.00 | 0 | 0.00 |
| Weddell | InTrough | Max | 0.00 | 0 | 0.00 |
| Weddell | InTrough | RF | 0.00 | 0 | 0.00 |
| Weddell | meanbathy | Max | 0.03 | 0 | 0.00 |
| Weddell | meanbathy | RF | 0.04 | 0 | 0.00 |
| Weddell | meanbathy | BRT | 0.05 | 0 | 0.00 |
| Weddell | meanslope | BRT | 0.05 | 0 | 0.00 |
| Weddell | meanslope | Max | 0.01 | 0 | 0.00 |
| Weddell | meanslope | RF | 0.03 | 0 | 0.00 |
| Weddell | Persistence2Years | BRT | 0.01 | 3 | 0.02 |
| Weddell | Persistence2Years | Max | 0.04 | 3 | 0.11 |
| Weddell | Persistence2Years | RF | 0.03 | 3 | 0.08 |
| Weddell | PredictabilityDec5Years | BRT | 0.05 | 3 | 0.16 |
| Weddell | PredictabilityDec5Years | Max | 0.00 | 3 | 0.00 |
| Weddell | PredictabilityDec5Years | RF | 0.06 | 3 | 0.18 |
| Weddell | PredictabilityOct5Years | BRT | 0.02 | 3 | 0.06 |
| Weddell | PredictabilityOct5Years | Max | 0.06 | 3 | 0.17 |
| Weddell | PredictabilityOct5Years | RF | 0.03 | 3 | 0.10 |
| crabeater | bathy | RF | 0.13 | 2 | 0.25 |
| crabeater | bathy | BRT | 0.07 | 2 | 0.13 |
| crabeater | bathy | Max | 0.14 | 2 | 0.29 |
| crabeater | dist_canyon | BRT | 0.05 | 1 | 0.05 |
| crabeater | dist_canyon | RF | 0.07 | 1 | 0.07 |
| crabeater | dist_canyon | Max | 0.05 | 1 | 0.05 |
| crabeater | dist_shelf | RF | 0.10 | 2 | 0.20 |
| crabeater | dist_shelf | Max | 0.02 | 2 | 0.04 |
| crabeater | dist_shelf | BRT | 0.04 | 2 | 0.09 |
| crabeater | ice_edge_dist | RF | 0.14 | 3 | 0.41 |
| crabeater | ice_edge_dist | Max | 0.11 | 3 | 0.32 |
| crabeater | ice_edge_dist | BRT | 0.28 | 3 | 0.83 |
| crabeater | ice_sd | RF | 0.10 | 3 | 0.29 |
| crabeater | ice_sd | Max | 0.10 | 3 | 0.29 |
| crabeater | ice_sd | BRT | 0.14 | 3 | 0.42 |
| crabeater | oldice_cv | Max | 0.06 | 2 | 0.11 |
| crabeater | oldice_cv | BRT | 0.04 | 2 | 0.09 |
| crabeater | oldice_cv | RF | 0.05 | 2 | 0.11 |
| crabeater | sal200_600 | RF | 0.07 | 1 | 0.07 |
| crabeater | sal200_600 | BRT | 0.06 | 1 | 0.06 |
| crabeater | sal200_600 | Max | 0.10 | 1 | 0.10 |
| crabeater | shflux_sd | RF | 0.09 | 2 | 0.19 |
| crabeater | shflux_sd | RF | 0.09 | 2 | 0.19 |
| crabeater | shflux_sd | Max | 0.02 | 2 | 0.04 |
| crabeater | shflux_sd | Max | 0.02 | 2 | 0.04 |
| crabeater | shflux_sd | BRT | 0.03 | 2 | 0.06 |
| crabeater | shflux_sd | BRT | 0.03 | 2 | 0.06 |
| crabeater | slope | RF | 0.00 | 0 | 0.00 |
| crabeater | slope | Max | 0.05 | 0 | 0.00 |
| crabeater | slope | BRT | 0.05 | 0 | 0.00 |
| crabeater | sst | RF | 0.03 | 3 | 0.09 |
| crabeater | sst | Max | 0.15 | 3 | 0.46 |
| crabeater | sst | BRT | 0.03 | 3 | 0.10 |
| crabeater | vmix | RF | 0.07 | 2 | 0.14 |
| crabeater | vmix | Max | 0.06 | 2 | 0.12 |
| crabeater | vmix | BRT | 0.04 | 2 | 0.07 |
| crabeater | vmix_sd | Max | 0.07 | 1 | 0.07 |
| crabeater | vmix_sd | RF | 0.05 | 1 | 0.05 |
| crabeater | vmix_sd | BRT | 0.03 | 1 | 0.03 |
| crabeater | windmag | BRT | 0.07 | 2 | 0.14 |
| crabeater | windmag | Max | 0.05 | 2 | 0.10 |
| crabeater | windmag | RF | 0.06 | 2 | 0.13 |


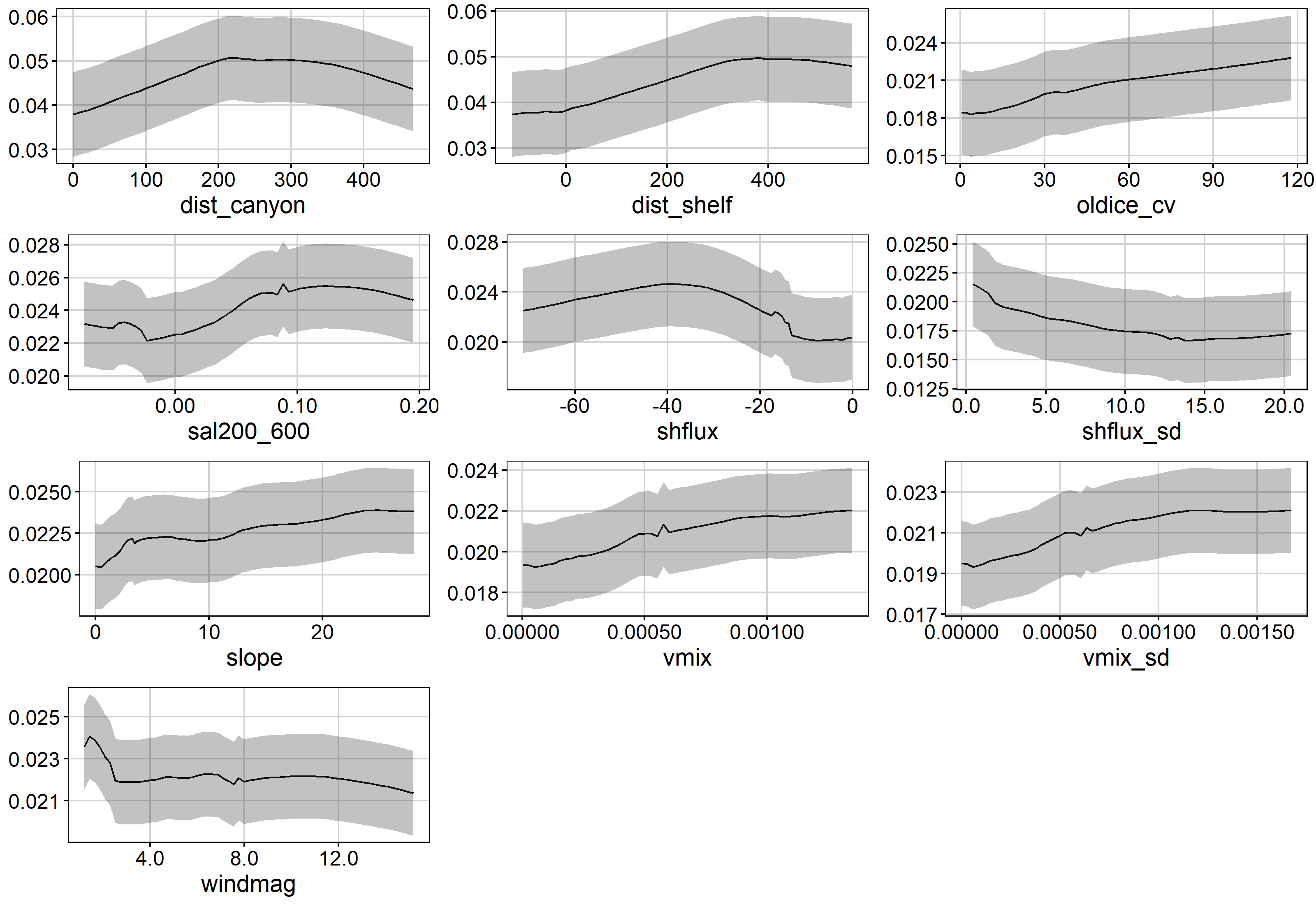


Figure A1: Partial dependence plots showing the relationship between the probability of crabeater seal (*Lobodon carcinophaga*) presence (± standard deviation across the models and bootstraps) and the remaining environmental variables that did not fall under the top 4.


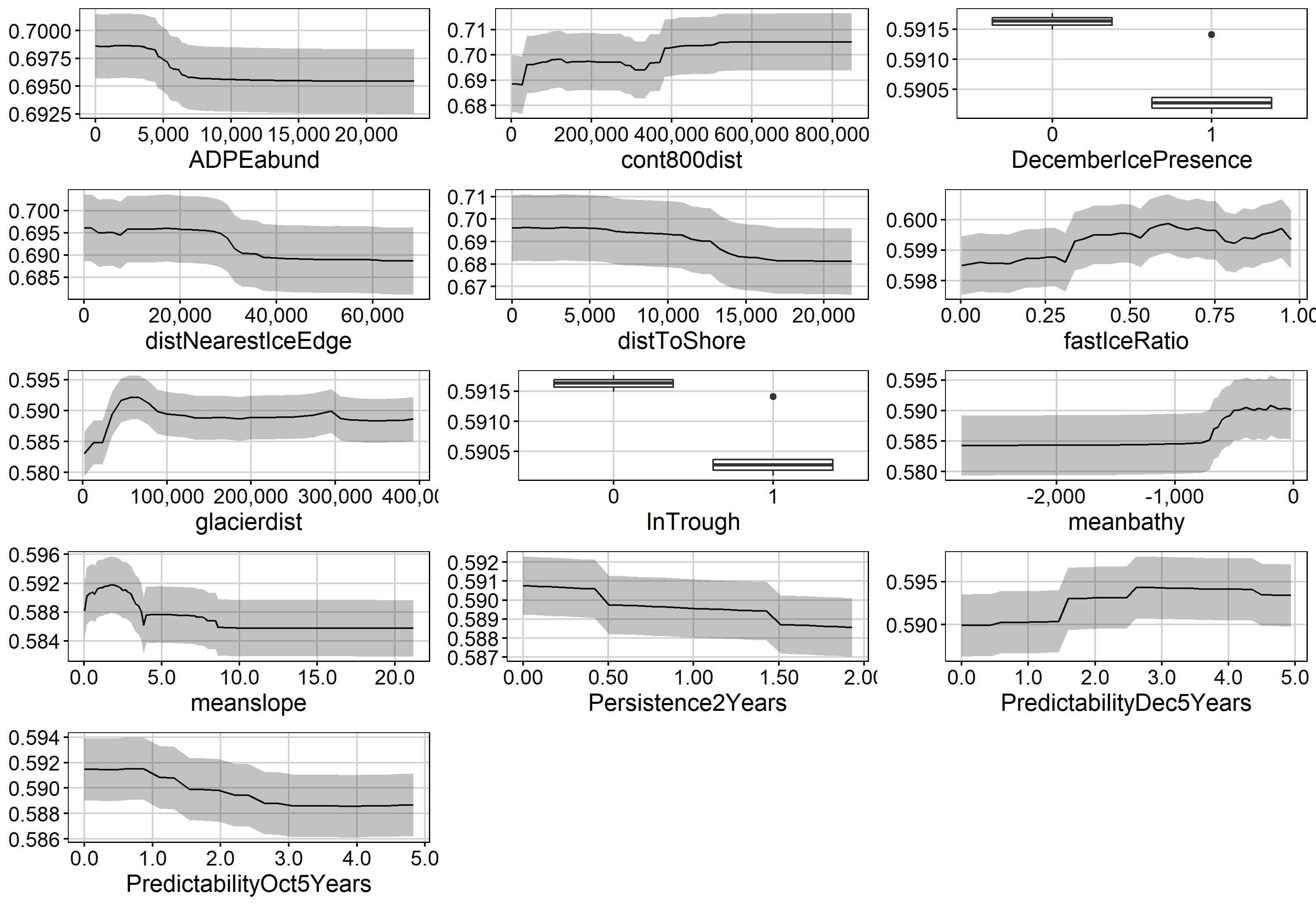


Figure A2: Partial dependence plots showing the relationship between the probability of Weddell seal (*Leptonychotes weddellii*) presence (± standard deviation across the models and bootstraps) and the remaining environmental variables that did not fall under the top 4.

**Literature Cited:**

Ainley, D. G., Ballard, G., Jones, R. M., Jongsomjit, D., Pierce, S. D., Jr, W. O. S., & Veloz, S. (2015). Trophic cascades in the western ross sea, antarctica: Revisited. *Marine Ecology Progress Series*, *534*, 1–16. https://doi.org/10.3354/meps11394

Fretwell, P. T., Trathan, P. N., Wienecke, B., & Kooyman, G. L. (2014). Emperor penguins breeding on iceshelves. *PLoS ONE*, *9*(1), 1–9. https://doi.org/10.1371/journal.pone.0085285

Lynch, H. J., & LaRue, M. A. (2014). First global census of the Adélie Penguin. *The Auk*, *131*(4), 457–466. https://doi.org/10.1642/AUK-14-31.1
